# Supplementary figures and images for: Testing for association with rare variants in the coding and non-coding genome: RAVA-FIRST, a new approach based on CADD deleteriousness score
Source: PLoS Genet. 2022 Sep 16;18(9):e1009923. doi: 10.1371/journal.pgen.1009923 (PMC9518893; doi:10.1371/journal.pgen.1009923)

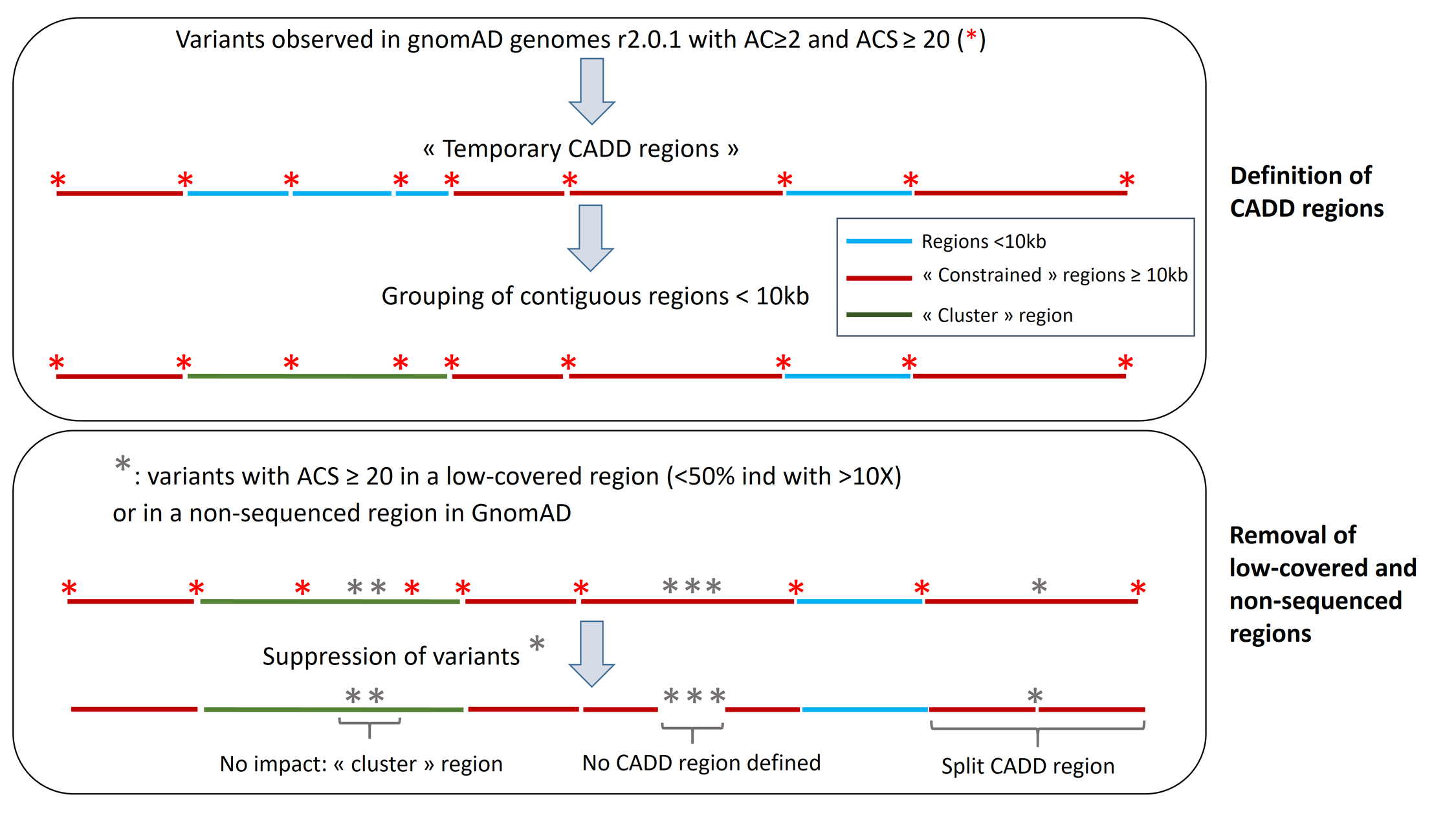

Supplement: S1 Fig — (TIF) [file pgen.1009923.s001.tif]

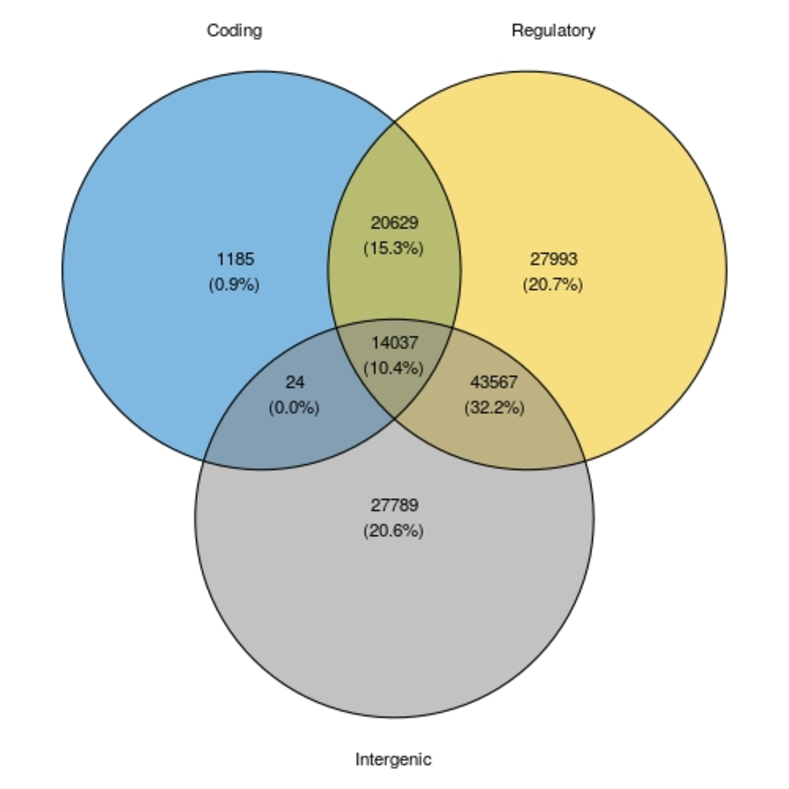

Supplement: S2 Fig — (TIF) [file pgen.1009923.s002.tif]

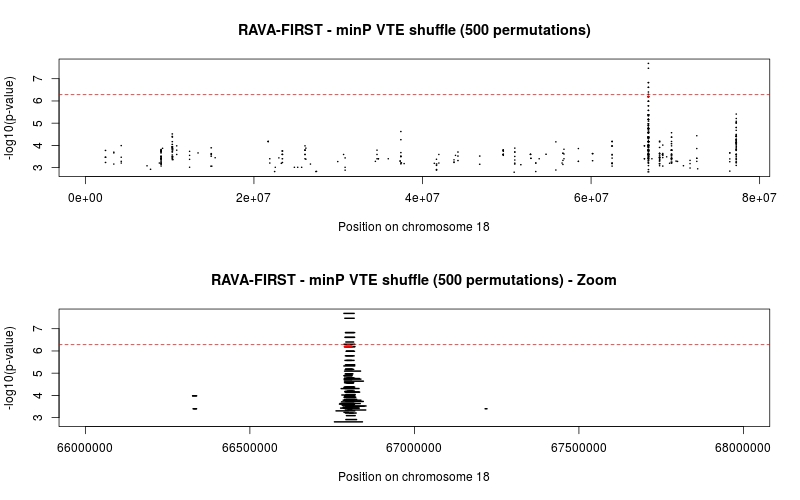

Supplement: S3 Fig — (TIF) [file pgen.1009923.s003.tif]

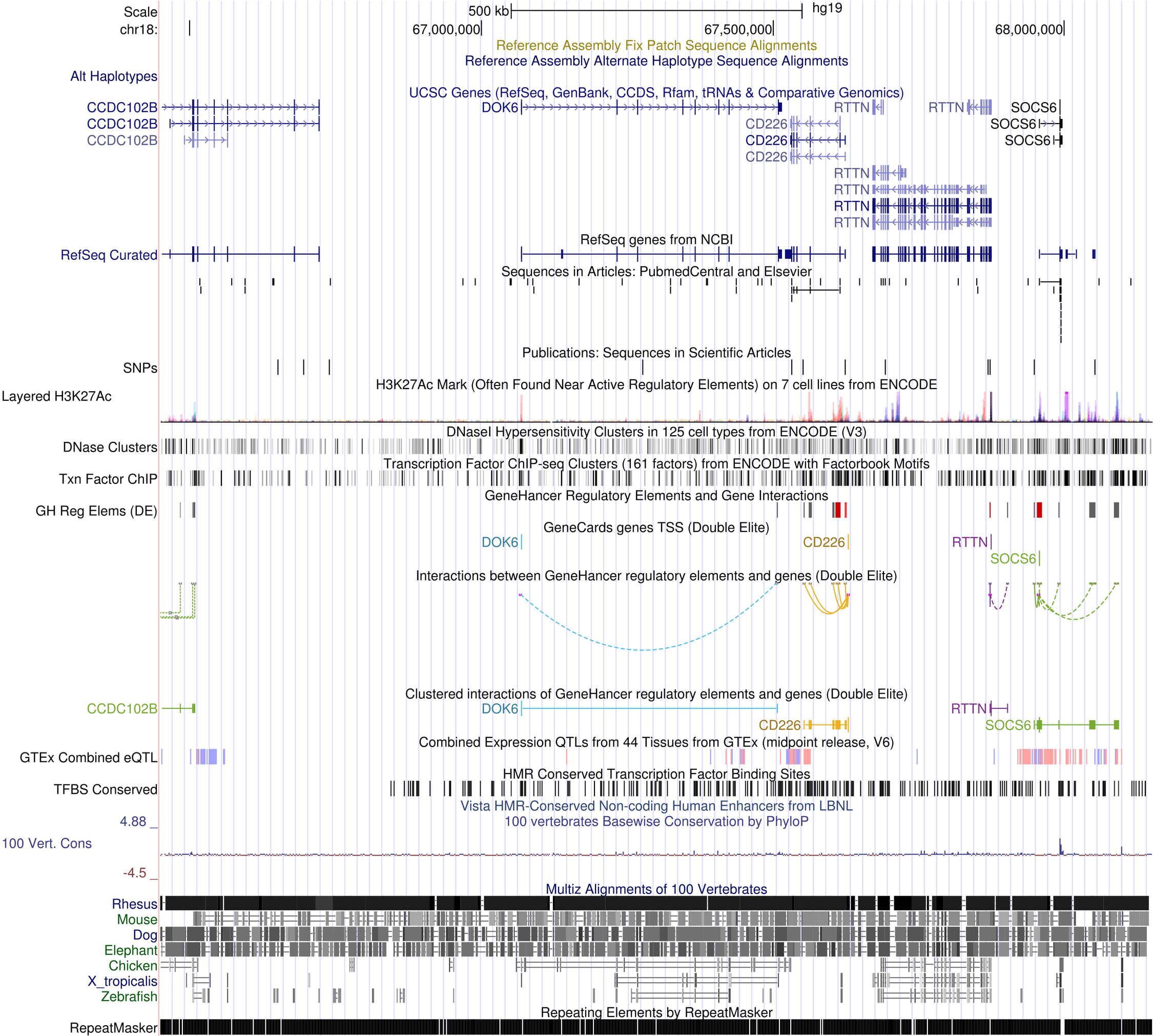

Supplement: S4 Fig — (TIF) [file pgen.1009923.s004.tif]
