# Supplementary material for: Testing for association with rare variants in the coding and non-coding genome: RAVA-FIRST, a new approach based on CADD deleteriousness score
Source: PLoS Genet. 2022 Sep 16;18(9):e1009923. doi: 10.1371/journal.pgen.1009923 (PMC9518893; doi:10.1371/journal.pgen.1009923)
Supplement: S1 Table — (DOCX) [file pgen.1009923.s005.docx]

S1 Table: Sources used to get genomic elements for comparisons with CADD regions

| **Genomic elements** | **URL** | **Details** |
| --- | --- | --- |
| CCDS | As for CADD regions definition | |
| Domains | <https://genomebiology.biomedcentral.com/articles/10.1186/s13059-016-0869-4> [1] | Additional file 10 |
| CCR | <https://github.com/quinlan-lab/ccr> [2] | Autosomal CCR regions in extended BED format (gnomAD v2.0.1) |
| Introns/UTR | As for CADD regions definition | |
| Enh-Prom DECRES | <https://bmcbioinformatics.biomedcentral.com/articles/10.1186/s12859-018-2187-1> [3] | Additional file 2 |
| Enh-Prom ENCODE | As for CADD regions definition | |
| Silencers | <http://health.tsinghua.edu.cn/silencerdb/download.php> [4] |  |
| CTCF | <https://screen.encodeproject.org/> [5] |  |
| lncRNA | <https://lncipedia.org/download> [6] | v5.2 |

# Reference

1. Gussow AB, Petrovski S, Wang Q, Allen AS, Goldstein DB (2016) The intolerance to functional genetic variation of protein domains predicts the localization of pathogenic mutations within genes. Genome Biol 17:9

2. Havrilla JM, Pedersen BS, Layer RM, Quinlan AR (2019) A map of constrained coding regions in the human genome. Nature Genetics 51:88–95

3. Li Y, Shi W, Wasserman WW (2018) Genome-wide prediction of cis-regulatory regions using supervised deep learning methods. BMC Bioinformatics 19:202

4. Zeng W, Chen S, Cui X, Chen X, Gao Z, Jiang R (2021) SilencerDB: a comprehensive database of silencers. Nucleic Acids Research 49:D221–D228

5. Moore JE, Purcaro MJ, Pratt HE, Epstein CB, Shoresh N, Adrian J, et al (2020) Expanded encyclopaedias of DNA elements in the human and mouse genomes. Nature 583:699–710

6. Volders P-J, Anckaert J, Verheggen K, Nuytens J, Martens L, Mestdagh P, Vandesompele J (2019) LNCipedia 5: towards a reference set of human long non-coding RNAs. Nucleic Acids Research 47:D135–D139
