# Supplementary material for: Testing for association with rare variants in the coding and non-coding genome: RAVA-FIRST, a new approach based on CADD deleteriousness score
Source: PLoS Genet. 2022 Sep 16;18(9):e1009923. doi: 10.1371/journal.pgen.1009923 (PMC9518893; doi:10.1371/journal.pgen.1009923)
Supplement: S2 Table — (DOCX) [file pgen.1009923.s006.docx]

S2 Table: Percentage of genomic elements entirely encompassed within a CADD region

| Exon CCDS | Protein domains | CCR | Introns/UTR | Enh-Prom | | Silencers | CTCF | lncRNA |
| --- | --- | --- | --- | --- | --- | --- | --- | --- |
|  |  |  |  | DECRES | ENCODE |  |  |  |
| 97.8% | 81.8% | 99.2% | 85.9% | 93.1% | 96.4% | 95.1% | 95.8% | 65.5% |
