# Supplementary material for: Testing for association with rare variants in the coding and non-coding genome: RAVA-FIRST, a new approach based on CADD deleteriousness score
Source: PLoS Genet. 2022 Sep 16;18(9):e1009923. doi: 10.1371/journal.pgen.1009923 (PMC9518893; doi:10.1371/journal.pgen.1009923)
Supplement: S3 Table — (DOCX) [file pgen.1009923.s007.docx]

S3 Table: Type I error of the classical WSS and the RAVA-FIRST WSS using 5∙10^6^ simulations under the null hypothesis

|  | $\alpha=0.05$ | $\alpha={10}^{-3}$ | $\alpha=2.5\cdot{10}^{-6}$ |
| --- | --- | --- | --- |
| Classical WSS | $0.051$ | $1.04\cdot{10}^{-3}$ | $3.02\cdot{10}^{-6}$ |
| RAVA-FIRST WSS | $0.051$ | $1.05\cdot{10}^{-3}$ | $2.80\cdot{10}^{-6}$ |
