# Supplementary material for: Testing for association with rare variants in the coding and non-coding genome: RAVA-FIRST, a new approach based on CADD deleteriousness score
Source: PLoS Genet. 2022 Sep 16;18(9):e1009923. doi: 10.1371/journal.pgen.1009923 (PMC9518893; doi:10.1371/journal.pgen.1009923)
Supplement: S4 Table — Mean (Standard Deviation) for quantitative variables. Count (%) for qualitative variables. (DOCX) [file pgen.1009923.s008.docx]

S4 Table: Characteristics of the studied VTE sample. Mean (Standard Deviation) for quantitative variables. Count (%) for qualitative variables

| Variables | Late Onset VTE | Early Onset VTE | |
| --- | --- | --- | --- |
|  |  | Null WSS score | Non-null WSS score |
| N | 54 | 100 | 30 |
| Age at first VTE | 61.5 (7.7) | 34.2 (9.2) | 34.4 (8.7) |
| Age at sampling | 63.2 (7.6) | 39.4 (11.1) | 40.0 (10.1) |
| **Sex** |  |  |  |
| Male | 21 (39%) | 23 (23%) | 4 (13%) |
| Female | 33 (61%) | 77 (77%) | 26 (87%) |
| **Type of first VTE** |  |  |  |
| Deep Vein Thrombosis | 40 (74%) | 83 (83%) | 25 (83%) |
| Pulmonary Embolism | 14 (26%) | 17 (17%) | 5 (17%) |
| BMI | 27.1 (3.8) | 24.6 (4.6) | 25.9 (5.1) |
| Hematocrit | 0.42 (0.03) | 0.41 (0.03) | 0.38 (0.03) |
| Red blood cells count | 4.77 (0.36) | 4.58 (0.37) | 4.38 (0.32) |
