# Supplementary material for: Testing for association with rare variants in the coding and non-coding genome: RAVA-FIRST, a new approach based on CADD deleteriousness score
Source: PLoS Genet. 2022 Sep 16;18(9):e1009923. doi: 10.1371/journal.pgen.1009923 (PMC9518893; doi:10.1371/journal.pgen.1009923)
Supplement: S1 File — (DOCX) [file pgen.1009923.s009.docx]

# Supporting information

## CADD regions

CADD regions were defined for autosomes only after computing adjusted CADD scores (ACS) for SNVs and Indels as described below.

### Adjusted CADD scores

#### SNVs

Adjusted CADD scores (ACS) were derived from GRCh37 RAW CADD scores v1.4^1^. Three genomic categories were defined to adjust these RAW scores: (1) coding regions were defined as coding parts of exons, obtained from BioMart ENSEMBL GRCh37 by selecting “genomic coding start” and “genomic coding end” attributes for all autosomal protein-coding transcripts from protein-coding genes. (2) Regulatory regions were defined as the union of UTR, introns, promoters and enhancers. UTR and introns were defined as the difference between gene positions using the same BioMart^2^ query and the previously defined coding regions. Promoters and enhancers were defined by the ENCODE project^3^ and downloaded from the SCREEN tool (<https://screen.encodeproject.org/>). The liftover tool from UCSC^4^ (<http://genome.ucsc.edu/cgi-bin/hgLiftOver>) was used to convert these positions to GRCh37 build. (3) Intergenic categories were defined as the rest of the genome being neither coding nor regulatory. After these three genomic categories were defined, CADD RAW scores v1.4 were PHRED-scaled for each substitution *i* among the *P* substitution within each genomic category as:

$${Adjusted CADD}_{i}={-10\cdot log}_{10}\left( \frac{\sum_{p=1}^{P} I({RAW}_{p}\geq{RAW}_{i})}{P} \right)$$

#### InDels

A similar approach was applied to InDels with the only difference that, unlike SNVs, all possible InDels are not available. To obtain InDels ACS, we therefore adjusted the CADD scores of a set of 48,000,000 variants provided by CADD (<https://krishna.gs.washington.edu/download/CADD/v1.4/GRCh37/InDels.tsv.gz>). When an InDel not observed in this set is provided to Ravages, its PHRED1.4 CADD score (computed using the online tool (<https://cadd.gs.washington.edu/score>) is compared to the PHRED1.4 CADD scores of InDels in the corresponding genomic area from the initial set of 48,000,000 InDels and is given the ACS of the InDel with the closest PHRED1.4.

### Definition of CADD regions

CADD regions were defined by selecting variants with an ACS greater than 20 observed at least two times in gnomAD genomes r2.0.1^5^. Temporary regions were obtained by using these variants as regions boundaries. Contiguous regions shorter than 10kb were then grouped together to form clusters of variants with high ACS. Low-covered regions and non-sequenced regions in gnomAD genomes were taken into account as followed: (1) Low-covered regions were defined as regions where less than 50% of individuals have a coverage greater than 10; (2) variants within these low-covered regions or within non-sequenced regions with an ACS greater than 20 were selected; (3) only such variants in regions larger than 10kb were kept; (4) these variants were removed from the CADD regions. One CADD region can therefore be split into two regions and gaps between CADD regions can be observed if multiple low-covered variants with high ACS are present (no CADD region is defined in these gaps). All these steps are summarised in Figure S1.

## Evaluation of adjusted CADD scores

### Selection of variants

To evaluate the performance of ACS, we downloaded the Clinvar^6^ vcf clinvar_20210731.vcf.gz from <https://ftp.ncbi.nlm.nih.gov/pub/clinvar/vcf_GRCh37/>. We selected only autosomal variants that were predicted “Pathogenic” and restricted the analysis to SNVs. As we were interested in RVAT, we kept only variants that had a minor allele frequency lower than 1% in each of the five gnomAD Genomes populations r2.0.1. For the “benign” dataset, we considered rare polymorphisms (frequency lower than 1%) observed in the 1000Genomes project^7^ and sampled a similar number of variants as observed in the Pathogenic dataset. The variants selected for the comparisons are available in the S2 File.

### Annotation of the variants

We annotated the selected variants with CADD scores v1.4 and the ACS. We also attributed a CADD region to each variant and get the corresponding median computed on ACS of variants observed at least two times in gnomAD. We further annotated variants with VEP^8^ v91 to get their consequence on the corresponding genes.

#### Coding variants

Among the observed annotations in the analysed dataset, we selected coding variants as variants having an annotation among “missense_variant”, “missense_variant&splice_region_variant”, “splice_acceptor_variant”, “splice_donor_variant”, “start_lost”, “start_lost&splice_region_variant”, “stop_gained”, “stop_gained&splice_region_variant”, “stop_lost”, “stop_lost&splice_region_variant” and “stop_retained_variant”, “splice_region_variant&synonymous_variant”, “synonymous_variant”. To compare our filtering strategy with a filtering based on the functional consequences of the variants, we considered the annotations “missense_variant”, “missense_variant&splice_region_variant”, “splice_acceptor_variant”, “splice_donor_variant”, “start_lost”, “start_lost&splice_region_variant”, “stop_gained”, “stop_gained&splice_region_variant”, “stop_lost”, “stop_lost&splice_region_variant” and “stop_retained_variant” as being at least missense. Finally, we used a filtering approach based on the MSC score for coding variants by first retrieving the gene of each variant from the vep annotation and then the corresponding MSC score using the online tool <http://pec630.rockefeller.edu:8080/MSC/>. To apply this MSC threshold by gene, CADD score v1.3 were retrieved for each variant as this version was used by the authors to compute the MSC. For the analysis on the coding genome, variants in genes without a MSC score were removed from the analysis.

#### Non-coding variants

In addition, the evaluation of the ACS was also done on the non-coding variants simply defined as all variants not having the previously listed coding annotations.

### Calculation of parameters

We computed the true positive rate (TPR), the true negative rate (TNR) and the precision for each filtering strategy based on the following table:

|  | Score < Threshold | Score ≥ Threshold |
| --- | --- | --- |
| Benign | TN | FP |
| Pathogenic | FN | TP |

The TPR was computed as $\frac{TP}{TP+FN}$; the TNR as $\frac{TN}{TN+FP}$ and the precision as $\frac{TP+TN}{TP+TN+FP+FN}$.

# References

1 Rentzsch, P., Witten, D., Cooper, G. M., Shendure, J. & Kircher, M. CADD: predicting the deleteriousness of variants throughout the human genome. *Nucleic Acids Res* **47**, D886-D894, doi:10.1093/nar/gky1016 (2019).

2 Durinck, S., Spellman, P. T., Birney, E. & Huber, W. Mapping identifiers for the integration of genomic datasets with the R/Bioconductor package biomaRt. *Nat Protoc* **4**, 1184-1191, doi:10.1038/nprot.2009.97 (2009).

3 Consortium, E. P. *et al.* Expanded encyclopaedias of DNA elements in the human and mouse genomes. *Nature* **583**, 699-710, doi:10.1038/s41586-020-2493-4 (2020).

4 Kent, W. J. *et al.* The human genome browser at UCSC. *Genome Res* **12**, 996-1006, doi:10.1101/gr.229102 (2002).

5 Karczewski, K. J. *et al.* The mutational constraint spectrum quantified from variation in 141,456 humans. *Nature* **581**, 434-443, doi:10.1038/s41586-020-2308-7 (2020).

6 Landrum, M. J. *et al.* ClinVar: improving access to variant interpretations and supporting evidence. *Nucleic Acids Res* **46**, D1062-D1067, doi:10.1093/nar/gkx1153 (2018).

7 Genomes Project, C. *et al.* A global reference for human genetic variation. *Nature* **526**, 68-74, doi:10.1038/nature15393 (2015).

8 McLaren, W. *et al.* The Ensembl Variant Effect Predictor. *Genome Biol* **17**, 122, doi:10.1186/s13059-016-0974-4 (2016).
