# Supplementary material for: Testing for association with rare variants in the coding and non-coding genome: RAVA-FIRST, a new approach based on CADD deleteriousness score
Source: PLoS Genet. 2022 Sep 16;18(9):e1009923. doi: 10.1371/journal.pgen.1009923 (PMC9518893; doi:10.1371/journal.pgen.1009923)
Supplement: Attachment — Submitted filename: Reviews_PlosG_23May22_VF.docx [file pgen.1009923.s012.docx]

**Reviewer #1: In their publication 'Testing for association with rare variants in the coding and non-coding genome: RAVA-FIRST, a new approach based on CADD deleteriousness score', Bocher et. all describe a novel method for selecting disease-candidate variants among rare SNVs genome-wide. The described methodology is very interesting and enables new insights especially in previously less regarding genomic regions. I would recommend however to revise the text somewhat in order to improve readability:**

We would like to thank Reviewer 1 for all his constructive suggestions to improve the manuscript.

**Major:**

**- I would recommend to define the adjusted CADD scores once and then stick to that. Maybe use an acronym (e.g. ACS (adjusted CADD score), RAVA-Score or something like that). In my eyes, the switches between adjusted and not-adjusted CADD scores (i.e. l. 154) make the manuscript hard to read, especially as you define the adjustment multiple times. Something similar may help to distinguish 'all' and '>1000bp' CADD regions**

We agree that the readability of the manuscript could be improved by using acronyms. The proposed notation ACS has been adopted and refers now to the adjusted CADD score throughout the manuscript.

Regarding the sizes of the regions, we realized that the distinction between all CADD regions and those larger than 1kb was not useful and only confusing. Indeed, in real data analysis, the <1kb CADD regions can be used as testing units. There is only a risk that there might not be enough variants in these regions and thus that the power to detect an effect in these regions will be low. In this new version of the manuscript, we have no longer made this distinction based on the size of the regions.

**- Table 2: Are percentages only from within the chosen (>1000bp) CADD regions or all. Or why else are some coding CCDS not in a CADD region? Why are protein domains so much less covered (likelihood that entire domain is covered instead of % of each bp)?**

We apologize for the lack of clarity. Table 2 refers to all CADD regions. As stated earlier, all statistics are now shown for all CADD regions. Table 2 shows the percentage of each genomic element entirely encompassed within a CADD region. Therefore, if a CCDS is overlapped by more than one CADD region, it will not be present in this table. The genomic regions encoding protein domains can be separated by large introns which decreases the probability that they are entirely encompassed within a CADD region and explains the lower percentage values observed in Table 2 for these elements.

**- Fig 1: Why do you include missense and MSC in the top panel. I understand that those are the same as the bottom panel, but the empty plots should hence be excluded altogether. Maybe you could use that space on the upper panel to put (a single) legend for both panels there (i.e. the TPR, TNR and precision colours).**

**One should note that Figure 1 does not tell much as most variants in ClinVar are coding and thus the adjusted CADD score does not do much. You mostly just have a higher cut-off than CADD1.4=20 (similar S4, just lower) which decreases TPR and increases FPR**

We agree that Figure 1 was not the best way of showing the performance of ACS. We have now modified it. The new Figure (which now corresponds to Figure 2) shows the performance for coding variants (top panel) and non-coding variants (bottom panel). We have modified the legend as suggested and removed Figure S4.

The ACS filtering performs better than the different fixed CADD thresholds, especially for coding variants. The ACS thresholds are different for each CADD region and it is therefore difficult to compare them to a hard cut-off like 20. Actually, as the scores and the median are adjusted on the three genomic areas, the cut-off used in each region are by far lower than 20.

**suggestion:**

**- I wonder what happens in the very large 'CADD regions' around the centromers where, presumedly, many variants are not scored at all and general genome conservation is low and hence the median, which may lead to small numbers of variants in slightly higher conserved areas to be considered significant, idk). I have no idea what effect this may have but I, personally, would probably implement a maximum length for CADD regions and split regions larger than, say, 200 kb into the maximum number so that each is at least 100 kb (just a suggestion, maybe this does not work as intended)**

The general conservation is indeed lower around the centromeres and thus CADD scores are low and this is indeed probably the reason why we observe such large regions around the centromeres. As you noted, CADD scores are not defined for variants in the centromeres and we have therefore not considered these areas when defining the CADD regions, meaning that there is no CADD region spanning over the centromeres. However, this does not solve the problem of these very large regions. Further work is probably needed to more precisely define regions of analysis around the centromeres but we are not keen to introduce a fixed size to split the regions around the centromeres as there would not be a biological hypothesis behind it. We have added some comments in the discussion to warn about this issue and ask investigators to be cautious if a signal is detected in these regions.

**- Maybe it's just me, but I find Figures S1 (to a lesser degree S2) definitely more relevant for the main manuscript than any of the Tables. You are generally moving a lot of the method (i.e. definition of CADD regions) in the supplement that seems an important part of the manuscript**

The choice of material included in the main text or in the supplementary data was indeed not the most relevant to illustrate RAVA-FIRST. We followed the reviewer’s advice and moved the Figure S1 to the main manuscript and Table 2 to the supplementary.

**- l. 320/321 'as recommended by https://cadd.gs.washington.edu/info, version v1.4': not to be pedantic but I would interpret 'there is not a natural choice here -- it is always arbitrary' rather as a recommendation for a dynamic threshold like your method than a single fixed cut-off. Afterall, you are pretty much proposing a (automated) solution for a problem that has also been stated there**

We agree and we have removed “recommended” and the website reference

**- l.156: ‘Note that because CADD scores are only available for SNVs’ -> CADD is available for InDel, consider carefully however if you want to include those in the analysis**

We agree that restricting the analysis to SNVs and not including InDel was a limit. As for SNVs, we observed that InDels show higher CADD scores in coding regions compared to regulatory and intergenic ones. We therefore had to adjust the InDel scores on the three genomic areas similarly to SNVs. As it is not feasible to evaluate every possible InDel in the genome as done for SNV, we used for this adjustment a set of 48,000,000 InDels already annotated in the CADD database (https://krishna.gs.washington.edu/download/CADD/v1.4/GRCh37/InDels.tsv.gz). In each CADD region, we computed the median of CADD values for both SNVs and InDels. We found a strong correlation between these medians and the ones computed using SNVs only. We therefore decided to offer a joint analysis of SNVs and InDels in RAVA-FIRST. All details have been added to the manuscript in the corresponding part.

Modifications of the R package Ravages to include InDels have also been made. As pre-defined scores for all InDels in the genome are not available, we ask the user to provide InDel PHRED1.4 scores. If the InDel to be annotated is present within the set of 48,000,000 InDels, it will be given the corresponding ACS. However, if the InDel to be annotated is not observed in this set, it will be given the ACS of the InDel with the closest PHRED1.4 CADD score in the corresponding genomic area in the set of 48,000,000 pre-computed scores.

ClinVar comparisons and VTE analysis in the manuscript are still performed on SNVs but the new median based on SNVs and InDels was used.

**minor:**

**- l. 89/90 'These regions prevent the use of sliding windows procedures while enabling the study of rare variants in the whole genome' -> I would use 'avoid' or 'replace' instead of 'prevent'**

**- l. 92 fix -> fixed**

**- (multiple) I assume 'package R Ravages' should be 'R package Ravages'**

**- (multiple) I would write 'gnomAD', not 'GnomAD'**

We thank the reviewers for pointing this out and we made the corresponding changes in the manuscript.

**Reviewer #2: In this manuscript, Bocher et al. attempt to define a new approach to performing rare variant burden tests in the non-coding genome. With whole-genome sequencing of large disease cohorts increasing at a rapid rate, identifying such methods is of value to the field. Unfortunately, the authors’ approach at defining functional units of the non-coding genome fails to account for major confounders. Furthermore, they have failed to benchmark against some of the most popular tools in the field as outlined in my comments below. In addition, their rationale for defining these CADD regions is very unclear to me.**

We thank the reviewer for his useful comments and we included various suggestions in this revised version of the manuscript. In particular, we clarified the aim of our study and how it differs from the one of Havrilla et al. The main goal of RAVA-FIRST is indeed not to find highly constrained regions in the genome but to propose a new strategy of analysis for rare variant association tests. We used a similar approach as Havrilla et al. to split the genome into regions based on the distribution of functional variants observed in gnomAD but the purpose was not to rank these regions based on a constraint. Therefore, CADD regions can show different levels of constraint and the differences between those regions is then further considered by applying a region-specific threshold to filter rare variants that depends on the distribution of scores that is observed in each region. We added a few changes highlighted in the manuscript to make the scope of RAVA-FIRST clearer to the reader.

**1. The authors define the boundaries of their CADD regions as regions between two variants with an adjusted CADD score > 20. However, they do not consider mutation rate. In CCR, which the authors compare their approach to, Havrilla et al. cleverly used CpG density as a proxy for mutation rate and showed that this approach worked well. Unfortunately, because the CADD regions do not account for mutation rate, it is entirely unclear whether the lack of variation in a CADD region is due to selective pressure or lower mutability of that region due to decreased mutation rate.**

We agree that for the purpose of ranking regions based on evolutionary constraints, as in Havrilla et al., it is important to takes into account CpG density. In our study however, as explained above, the objective is different as we only want to split the genome into regions to perform RVAT. For that purpose, we used the same idea as in Havrilla et al. to define the boundaries of regions but the comparison stops here. The differences of variant distribution within regions were then considered by computing a filtering threshold for each region based on the distribution of adjusted CADD scores in the general population. CADD regions are not expected to be used on their own but along with the associated filtering threshold. CpG density is also indirectly taken into account as it is one of the many features included in the construction of the CADD score.

**2. I’m not convinced that the authors are detecting regions of the genome depleted for functional variation. I’d like to see how their regions compare to other methods that attempt to define constrained genomic regions (JARVIS, Orion, CDTS, LINSIGHT, etc.). The authors should compare performance of these approaches in classifying non-coding ClinVar variants. Also, the authors should not use ClinVar benign variants as a negative set in these comparisons, as most ClinVar benign variants are gnomAD polymorphisms. Because gnomAD variation was used to define this score, they run into confounding with this comparison. A better approach would be to use variants seen in other databases (e.g., DiscovEHR) but not gnomAD as a putative benign set.**

As suggested by the reviewer, we compared CADD to JARVIS and LINSIGHT on non-coding variants as these are the only variants considered in LINSIGHT. We did not include Orion and CDTS in the ClinVar comparisons for the following reasons:

1. Orion has been shown to have a lower performance than CADD in different situations, especially in the study from Vitsios et al. 2021
2. CDTS scores are not position-specific and are computed for blocks of 10pb. We therefore think that this is not accurate enough to discriminate between rare variants within testing units.

Unexpectedly, we found a poor performance of JARVIS when comparing non-coding Clinvar variants to 1kG polymorphisms matched for minor allele frequencies. We hypothesized that this low performance could be due to the selection of rare variants (frequency lower than 1%) in these comparisons. LINSIGHT and CADD show similar prediction but as LINSIGHT is only available for the non-coding genome, we can’t use it for the RAVA-FIRST approach. We therefore decided to include only the CADD score for ClinVar comparisons based on these results. In addition, while all the other scores are position-specific, CADD scores are defined at an allele-specific level, which is an interesting feature for the selection of rare variants, especially multi-allelic ones. Finally, it is well-known that such score comparisons are very sensitive to the choice of the variant sets. We do not claim that the CADD score is the best in all situations, but it was found to perform the best or close to the best in a lot of different independent studies. Moreover, the interest here was mainly to investigate whether there was an interest of considering a variable threshold model rather than a fixed threshold model to select rare variants in RVAT. We have added some comments in the discussion regarding these other scores (see also our reply to the next comment).

We followed the reviewer’s advice and compared the ClinVar pathogenic set to another benign set by using the rare polymorphisms observed in the 1000Genomes project. The RAVA-FIRST strategy still retains its power over all the classical filtering strategies in the coding and the non-coding genome, especially through a higher TNR in the coding genome and a higher TPR in the non-coding genome.

**3. In defining CADD regions, the authors required the variant to be seen in gnomAD > 2x, but they do not provide any rationale how they came to this threshold. Same goes for choosing a CADD threshold for the burden tests.**

The rational for considering only gnomAD variants seen at least twice was indeed not given in the previous version of the manuscript. It was in fact to avoid singletons as a larger proportions of them are sequencing errors. We have now explained this choice. Concerning the CADD threshold to define CADD regions, we did try to use different score levels (10, 15, 25). We chose a threshold of 20 as it resulted in testing units that had the most compatible sizes with a classical rare variant analysis. For the burden tests, no hard threshold is used but rather a threshold that depends on the distribution of scores observed in the general population to solve the question of choosing a specific threshold. We did try to use the mean of the adjusted CADD score instead of the median within each CADD region but it resulted in a lower performance in discriminating pathogenic from benign ClinVar variants. We included this point in the discussion of the pros and cons of RAVA-FIRST at the end of the manuscript.

**4. While the authors compare to sliding-window based burden approaches, they should also compare to how their approach compares to defining functional units using the many other intolerance methods mentioned above.**

We did not perform such a comparison because we found that most of the other intolerance scores proposed in the literature were not as convenient and suited to be used in RVAT as CADD scores. Indeed, only few of them are able to predict intolerance on a variant-specific basis and to deal with both coding and non-coding variation. Our benchmarking of these scores presented above suggest that CADD scores perform among the best, especially in the non-coding genome. It would be possible, provided some adaptations of the proposed framework, to use other scores to define testing units in the same spirit as the proposed CADD regions. However, we think this is beyond the scope of this work as it will require some intensive work and important computing times to choose thresholds to ensure that testing units have a correct size and to compute adjusted scores like we did here with the adjusted CADD scores.

**5. They exclude indels/structural variants because these variants do not have CADD scores. Doing so results in a tremendous loss of power: indels / SVs should have much larger effect sizes than SNVs in the non-coding genome. A burden model that includes SNVs with a CADD threshold + any indels / SVs should be more powered.**

We agree that not considering the InDels in the analysis was a weakness of our approach and this point was also raised by the other reviewer. To answer this issue, we have implemented the analysis of InDels into the RAVA-FIRST pipeline in Ravages. As for SNVs, we observed that InDels show higher CADD scores in coding regions compared to regulatory and intergenic ones. We therefore had to adjust the InDel scores on the three genomic areas similarly to SNVs. As we do not have access to every possible InDel in the genome, we used a set of 48,000,000 InDels already annotated in the CADD database (https://krishna.gs.washington.edu/download/CADD/v1.4/GRCh37/InDels.tsv.gz). To offer the possibility to analyze InDels with RAVA-FIRST and apply the region-dependent filtering, we computed the median in each region by taking SNVs and InDel offering now the possibility to perform a joint analysis of SNVs and InDels in RAVA-FIRST. All details have been added to the manuscript in the corresponding part.

Modifications of Ravages to include InDels have also been made. As pre-defined scores for all InDels in the genome are not available, we ask the user to provide InDel scores. Furthermore, if an InDel is provided but absent from the set of 48,000,000 InDels used to adjust the score, it will be given the closest adjusted score in the corresponding genomic area.

ClinVar comparisons and VTE analysis in the manuscript are still performed on SNVs but the new median based on SNVs and InDels was used.

**6. The authors find a suggestive association between a CADD region and VTE. I’d like to see how their approach performs without any CADD filter as a negative control. Furthermore, I would want to see a comparison that their CADD region-based burden analysis does better than randomly splitting the genome into random chunks (matched in size to the CADD regions).**

We thank the reviewer for this suggestion and we have added these results in the new version of the manuscript. Using no CADD filter but only a MAF filter, we were able to detect the same top signal as before but the p-value was much higher (see Table 4 and Fig 3).

To compare our analysis to a random split of the genome, we analyzed the chromosome 18 where the top signal was found by shuffling the CADD regions 500 times. For each shuffle, we computed the new corresponding median in each region and performed the association analysis as before. When plotting the position of the region on the chromosome with the minimum p-value for each replicate (FigS3 in the new version of the manuscript), we see that it often encompasses the top signal identified in the original analysis. It is also only in this region that p-values reached the significance threshold. These results show that the partitioning of this chromosome with the proposed CADD regions improves power compared to random partitioning.
