# Supplementary material for: Testing for association with rare variants in the coding and non-coding genome: RAVA-FIRST, a new approach based on CADD deleteriousness score
Source: PLoS Genet. 2022 Sep 16;18(9):e1009923. doi: 10.1371/journal.pgen.1009923 (PMC9518893; doi:10.1371/journal.pgen.1009923)
Supplement: Attachment — Submitted filename: Review2_response_reviewers_2.docx [file pgen.1009923.s013.docx]

**Reviewer #1: All my previous concerns have been addressed. However, there are a few minor comments to the new sections that should be fixed prior to publication:**

We would like to thank Reviewer 1 for the minor comments that will improve the manuscript. All of them have been considered and the corresponding changes have been highlighted in the new version of the manuscript.

**l. 196/197: "This is expected given the fact that conservation and thus CADD scores are low in these regions."**

**l. 462/463 "We also observed that CADD regions close to the centromeres can be very large, possibly due to a general low conservation in these areas"**

**> My understanding is that there are few high quality genomes that cover these areas (repetitive elements, GC content) and hence alignments are missing to generate proper conservation scores.**

We agree that the prediction scores around the centromeres are probably not as reliable as in the rest of the genome due to fewer high-quality data in these areas. We made this point clear in l.198-200 and l.469 in the new version of the manuscript.

**l. 239 "Fig 2B" -> that is 2A now, right?**

**l. 477 "if" -> "of" (or the sentence does not make sense)**

**l. 479 "use of a" -> "use of the"**

We thank reviewer 1 for identifying these mistakes. They have been corrected in the updated version of the manuscript.

**optional:**

**Consider rereading and revising some sentences here and there. Quite a few feel unnecessarily lengthy.**

**E.g l. 478, you can remove "of them can" without changing context**

We followed the advice from reviewer 1 and alleviated multiple sentences to make the manuscript easier to read.
